# Histone variants and modifications during abiotic stress response

**DOI:** 10.3389/fpls.2022.984702

**Published:** 2022-12-15

**Authors:** Rocío Nunez-Vazquez, Bénédicte Desvoyes, Crisanto Gutierrez

**Affiliations:** Centro de Biología Molecular Severo Ochoa, CSIC-UAM, Programa de Dinámica y Función del Genoma, Madrid, Spain

**Keywords:** chromatin, epigenetics, histone, histone variant, histone modification, acetylation, methylation, abiotic stress

## Abstract

Plants have developed multiple mechanisms as an adaptive response to abiotic stresses, such as salinity, drought, heat, cold, and oxidative stress. Understanding these regulatory networks is critical for coping with the negative impact of abiotic stress on crop productivity worldwide and, eventually, for the rational design of strategies to improve plant performance. Plant alterations upon stress are driven by changes in transcriptional regulation, which rely on locus-specific changes in chromatin accessibility. This process encompasses post-translational modifications of histone proteins that alter the DNA-histones binding, the exchange of canonical histones by variants that modify chromatin conformation, and DNA methylation, which has an implication in the silencing and activation of hypervariable genes. Here, we review the current understanding of the role of the major epigenetic modifications during the abiotic stress response and discuss the intricate relationship among them.

## Introduction

Chromatin is a highly organized eukaryotic complex of DNA and proteins, where DNA is packaged into regularly spaced nucleosomes, assembled as beads on a string. Each nucleosome is formed by ∼147 bp of DNA wrapped around a core histone octamer (Olins and Olins, 1974; Thomas and Kornberg, 1975; Libertini et al., 1988; Luger et al., 1997; Wolffe and Hayes, 1999). Throughout evolution, histone proteins have gradually evolved from archaeal ancestors into the four distinct subunits that compose the common octamer of the nucleosome. The core histones H2A, H2B, H3, and H4 are structured in two H2A-H2B dimers and an H3-H4 tetramer. The linker histone H1 helps to condense the chromatin by binding to the DNA between nucleosomes (Campos and Reinberg, 2009; Henikoff and Smith, 2015; Talbert and Henikoff, 2017).

The chromatin landscape is in constant reorganization to guarantee the transcriptomic reprogramming required during developmental processes (Baulcombe and Dean, 2014; Kawashima and Berger, 2014; Xiao and Wagner, 2015; Lee and Seo, 2018; Gehring, 2019), such as germline differentiation (Borg et al., 2009; Feng et al., 2010; Baroux et al., 2011; Borg et al., 2021b) or leaf senescence (Brusslan et al., 2015). Alterations in chromatin structure have been associated to different states of DNA accessibility (Sequeira-Mendes et al., 2014). Functionally, chromatin is divided into two conformational states: heterochromatin, in which DNA is strongly condensed, and euchromatin, where the DNA is more accessible and less compacted. The molecular mechanisms regulating the switch between euchromatin and heterochromatin include complex epigenetic regulatory networks (Adam et al., 2001; Nakayama et al., 2001; Ahmad and Henikoff, 2002; Francis et al., 2004; Castellano-Pozo et al., 2013; Sequeira-Mendes et al., 2014; Yelagandula et al., 2014; Morrison and Thakur, 2021). We have included several excellent and recent reviews that discuss and detail the function of the major drivers of chromatin restructuration: histone variants (Loppin and Berger, 2020; Probst et al., 2020; Foroozani et al., 2022), histone post translational modifications (Antunez-Sanchez et al., 2020; Jiang et al., 2020b), and DNA methylation (Zhang et al., 2018; Mattei et al., 2022).

Abiotic factors such as salinity, limited water availability, extreme temperature, low-light and chemical composition of the soil severely impact plant growth and developmental programs. Thus, variations in any of these conditions lead to an alteration in the homeostasis, known as abiotic stress (Singh and Laxmi, 2015). Each form of abiotic stress contains a unique signaling pathway. Nevertheless, there are conserved cellular responses orchestrated by a complex regulatory network involving (1) upstream signaling molecules, such as ROS, NO, Ca^2+^ or ABA, and (2) downstream regulation, in which transcription factors and epigenetic regulators intervene (He et al., 2018). Here, we will focus on the downstream regulation and summarize the mechanisms of these epigenetic agents, which redefine the plant chromatin landscape when exposed to external stimuli.

**Figure 1:**
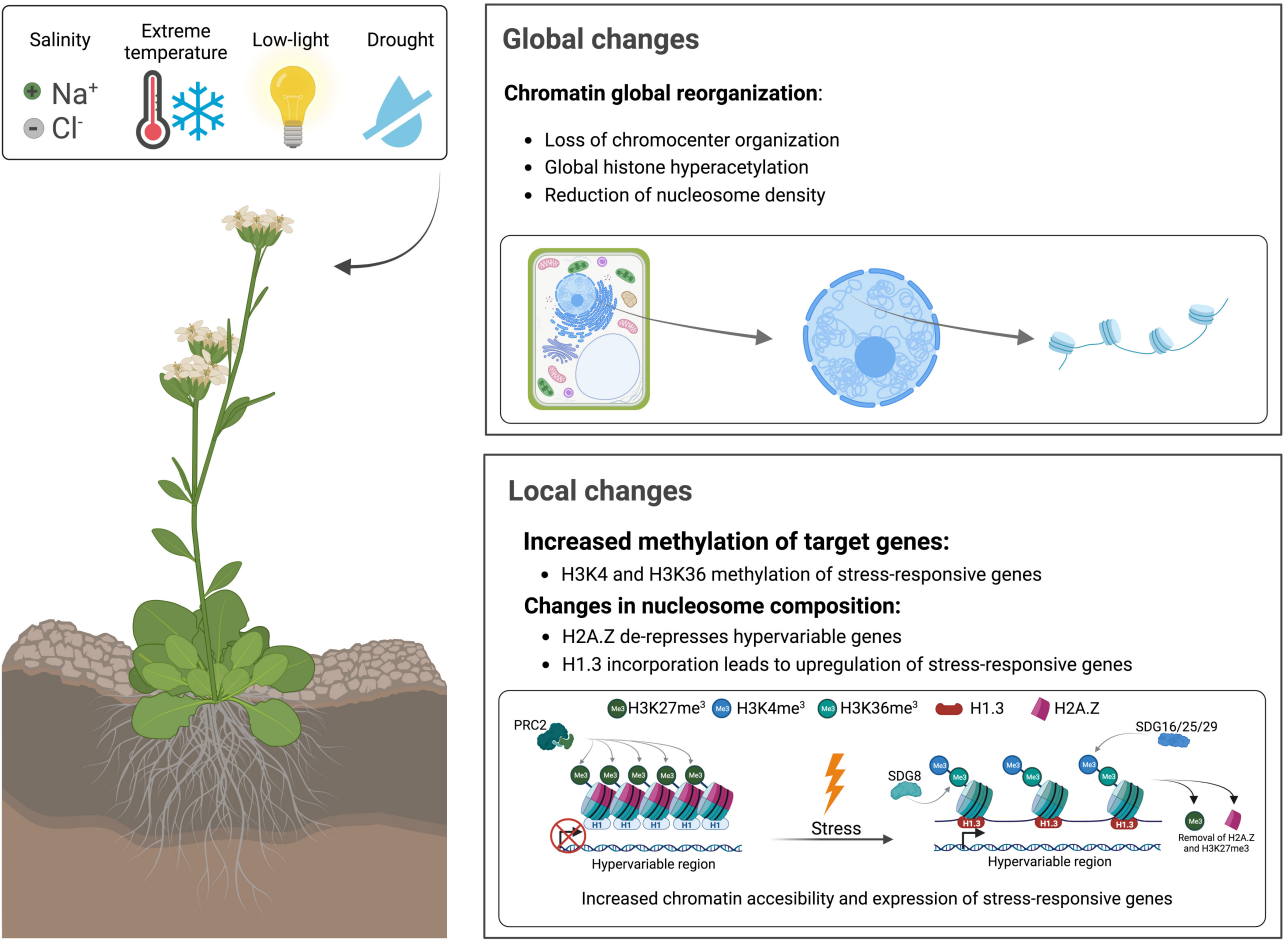
Global and local changes in chromatin structure in response to abiotic factors, such as salt, limited water availability, cold, heat, and low-light. Global changes are distributed genome-wide, whereas local changes are directed to specific genomic regions, commonly associated with stress-responsive genes.

There are different scales —global and local— at which these modifications happen in the context of abiotic stressors (**Figure 1**). Global changes in response to abiotic stress include an increase in histone acetylation (Pandey et al., 2002; Earley et al., 2007), a loss in the chromocenter organization —typical of plant heterochromatin— (Pecinka et al., 2010; reviewed in Probst and Mittelsten Scheid, 2015) and a reduction in nucleosome occupancy (Brzezinka et al., 2016; Park et al., 2018; reviewed in Bäurle and Trindade, 2020). These modifications occur globally in the sense that they are not directed to a particular genomic region but are genome-wide instead. On the contrary, there are local changes particular of stress-responsive areas of the genome characterized by an increase in methylation of the residues K4/K36 of H3 histone tails (Lee et al., 2016) and changes in nucleosome composition (Rutowicz et al., 2015). Additionally, there are gene-specific changes unique of each type of stress [e. g. *P5CS2* is upregulated upon salt stress and dehydration, whereas *HSP17.4* responds to heat (Port et al., 2004; Székely et al., 2008)]. However, their local epigenetic regulation shares identical features: an increase in histone modifications associated with an increase in DNA accessibility and a reduction in marks associated with less accessibility. During this review, we have decided not to focus on the specific changes that occur in presence of each abiotic factor, but instead on the general mechanisms involved in chromatin reorganization during the stress response —commonly shared between the different abiotic agents—. Deciphering how the expression of stress-responsive genes occurs is fundamental in unravelling the hidden details of the abiotic stress response.

How histone post translational modifications impact on the transcriptional changes required for plant survival during the stress response has been previously discussed (Kim et al., 2015; Ueda and Seki, 2020; Bhadouriya et al., 2021). However, the implication of histone variants during the abiotic stress response has not been discussed in depth. Moreover, most of the reviews about epigenetic regulation include either histone variants or histone modifications, but it is rare to see a combination of both. Hence, this review aims to furnish deeper insights into the transient coordination between histone variants and histone modifications in response to abiotic stress in plants.

*Arabidopsis thaliana* serves as an excellent model organism in plant research due to (1) its small, fully sequenced and well-annotated genome, (2) its short life cycle, (3) its tolerance to mutations in chromatin key genes, generally lethal in other organisms and (4) its post-embryonic organogenesis process characteristic of plants (Perianez-Rodriguez et al., 2014). These circumstances create an ideal scenario to study epigenetic changes during growth and morphogenesis in response to developmental and environmental cues. For that purpose, most of the epigenetic research in plants uses this organism as a model. In this review, we will focus on the major epigenetic modifications in the plant *Arabidopsis thaliana* as an approach to plant epigenetics.

## Histone variants

The paralogous genes of a histone family encode identical isoforms, but also non-allelic protein isoforms commonly referred to as histone variants. These variants differ in their amino acid sequence from the canonical form and play critical roles in diverse processes such as transcription, chromatin remodelling, and DNA packaging, conferring unique characteristics of chromatin (Talbert et al., 2012; reviewed in Talbert and Henikoff, 2017; Probst, 2022).

Canonical histones, also known as replicative histones, are predominantly expressed during the S-phase and deposited in a DNA synthesis-dependent manner. Conversely, histone variants, or replacement histones, are expressed throughout the cell cycle, are incorporated in a DNA synthesis-independent manner and have sequence divergence and specific genomic localization (Chaubet et al., 1992; reviewed in Henikoff and Smith, 2015).

Histone variants have been described in all model organisms studied, from the unicellular yeast *Saccharomyces cerevisiae* and algae to plants and animals, and for all histones but histone H4, with only a few exceptions (Long et al., 2019; reviewed in Probst et al., 2020). Some histone variants, like H3.3 and H2A.Z, are conserved in eukaryotes, while others are lineage-specific, such as the flowering plant-specific H2A.W variant (Yelagandula et al., 2014; Giaimo et al., 2019; Bourguet et al., 2021; Lei et al., 2021). There are tissue-specific variants, such as the *Arabidopsis* H3.10 and H2B.8, that function in sperm cells (Jiang et al., 2020a; Borg et al., 2021a; Buttress et al., 2022).

Given the important role that histone variants have in chromatin regulation, their deposition needs to be temporally orchestrated. Histone chaperones promote nucleosome assembly and disassembly during replication, transcription and repair (Daniel Ricketts et al., 2015; Hammond et al., 2017).

The diversity of nucleosome composition provided by canonical histones and variants is associated with different chromatin states. Depending on the histone variant incorporated into the nucleosome, chromatin adopts a more open —accessible to transcriptional machinery— or closed chromatin conformation. Thus, H3.3, H2A.Z, and H2A.X variants are abundant in euchromatic regions, along with histone marks in active chromatin, e.g., H3K4me3, H3K36me3 and H2B ubiquitylation, and coincide with high RNA Pol II occupancy (Stroud et al., 2012; Wollmann et al., 2012). These features form a chromatin state typical of active transcription (Sequeira-Mendes et al., 2014; Borg et al., 2021a). On the contrary, H2A.W and H1 histones colocalize with heterochromatin marks —like H3K9me2, H3K27me3, H3K27me1— and DNA methylation in silent genomic regions, favoring the compaction of the chromatin (Grewal and Jia, 2007; Vaillant and Paszkowski, 2007; Roudier et al., 2009; Stroud et al., 2012; Zemach et al., 2013; Rutowicz et al., 2019; Choi et al., 2020). The histone variants that play a role during the stress response are incorporated into nucleosomes in specific regions of the genome —stress-responsive genes— that are crucial for the upstream signaling stress response (Coleman-Derr and Zilberman, 2012; Rutowicz et al., 2015).

An intriguing feature of histone variants is the organization within one family. In histone families where there are several histone variant proteins with similar functions, there is usually a prevalence in the abundance of one or two among the others (reviewed in Martire and Banaszynski, 2020). This suggests either specific pathways ensure deposition of these variants or that the slight differences between the proteins lead to a favored deposition of some of them against the rest, developing into a specific role of certain variants. In *Arabidopsis*, the histones —canonical or variants— are organized into the four families (**Table 1**) discussed below.

**Table 1:** Classification of histone families in *Arabidopsis*: genes, variants, proteins, chaperones, general function and role in stress.

Histone type	Histone		Gene	Chaperone	General Function	Role in Stress
**H1**	H1.1	Variant	*At1g06760, H1.1*	NAP1, NRP1	Chromatin compaction (Bourguet et al., 2021)	
	H1.2	Variant	*At2g30620, H1.2*			
	H1.3	Variant	*At2g18050, H1.3*			Drought stress, light and water deficency (Rutowicz et al., 2019)
**H2A**	H2A.1	Canonical	*At5g54640, HTA1*	NAP1, NRP1, FACT		
	H2A.2	Canonical	*At4g27230, HTA2*			
	H2A.10	Canonical	*At1g51060, HTA10*			
	H2A.13	Canonical	*At3g20670, HTA13*			
		Variant	*At1g54690, HTA3*	FACT	Transcriptional activation (Xiao et al., 2021)	DNA damage response (Lorković and Berger, 2017)
		Variant	*At1g08880, HTA5*			
	H2A.W	Variant	*At5g59870, HTA6* *At5g27670, HTA7* *At5g02560, HTA12*	DDM1	Chromatin compaction (Bourguet et al., 2021)	DNA replication stress signalling in heterochromatin (Lorković and Berger, 2017)
		Variant				
		Variant				
	H2A.Z	Variant	*At4g13570, HTA4*	SWR1	Transcriptional regulation (Jarillo and Piñeiro, 2015) (Gómez-Zambrano et al., 2019)	Salt stress, drought, immunity responses, cold, heat, phosphate deficiency. Regulates the expression of hypervariable genes (Coleman-Derr and Zilberman, 2012) (Sura et al., 2017)
		Variant	*At2g38810, HTA8*			
		Variant	*At1g52740, HTA9*			
		Variant	*At3g54560, HTA11*			
**H2B**	H2B.1	Variant	*At1g07790, HTB1*	NAP1, NRP1, FACT	Transcriptional regulation, replacement variant (Jiang et al., 2020a)	
	H2B.2	Variant	*At5g22880, HTB2*			
	H2B.3	Variant	*At2g28720, HTB3*			
	H2B.4	Variant	*At5g59910, HTB4*			
	H2B.5	Variant	*At2g37470, HTB5*			
	H2B.6	Variant	*At3g53650, HTB6*			
	H2B.7	Variant	*At3g09480, HTB7*		Development of reproductive tissues (Jiang et al., 2020a)	
	H2B.8/H2B.S	Variant	*At1g08170, HTB8*		Regulation of seed formation (Jiang et al., 2020a)	
	H2B.9	Variant	*At3g45980, HTB9*			
	H2B.10	Variant	*At5g02570, HTB10*		Development of reproductive tissues (Jiang et al., 2020a)	
	H2B.11	Variant	*At3g46030, HTB11*			
**H3**	H3.1	Canonical	*At5g65360, HTR1*	CAF1	Transcriptional repression (Stroud et al., 2012; Wollmann et al., 2012)	
			*At1g09200, HTR2*			
			*At3g27360, HTR3*			
			*At5g10400, HTR9*			
			*At5g10390, HTR13*			
	H3.3	Variant	*At4g40030, HTR4* *At4g40040, HTR5* *At5g10980, HTR8*	HIRA, ATRX	Transcriptional repression (Stroud et al., 2012) (Wollmann et al., 2012)	Regulate hypervariable genes (Wollmann et al., 2012)
			
				
	H3.6	Variant	*At1g13370, HTR6*	HIRA?		Induced upon stress (Nunez-Vazquez et al., in preparation)
	H3.7	Variant	*At1g75610, HTR7*			
	H3.10	Variant	*At1g19890, HTR10*	HIRA?		
	H3.11	Variant	*At5g65350, HTR11*			
	CenH3	Variant	*At1g01370, HTR12*	HJURP		
	H3.14	Variant	*At1g75600, HTR14*	HIRA?		Induced upon stress (Nunez-Vazquez et al., in preparation)
	H3.15	Variant	*At5g12910, HTR15*	HIRA	Callus formation (Yan et al., 2020)	Rapidly induced upon wounding (Yan et al., 2020)
**H4**	H4	Canonical	*At3G46320*	CAF1, HIRA, ASF1	Mainteinance of genome integrity	
			*At5G59690*			
			*At2G28740*			
			*At1G07820*			
			*At3G53730*			
			*At5G59970*			
			*At3G45930*			
			*At1G07660*			

## H1 family

H1 histones are known as “linker histones” because they bind to the linker DNA between nucleosomes, further facilitating chromatin compaction. These histones consist of a globular domain, which binds the DNA at the dyad axis of the nucleosome to the core histones, a short N-terminal chain, and a C-terminal tail that binds to the DNA between nucleosomes (Zhou et al., 2013; Zhou et al., 2015). The tight bound between the nucleosome and the linker DNA results in higher nucleosome density. Histone H1, together with H2A.W, coordinates heterochromatin accessibility and DNA methylation (Bourguet et al., 2021). In vertebrates, several evolutionarily conserved subfamilies of H1 can be distinguished, and they play redundant and specific roles during development and cellular differentiation (Mcbryant et al., 2010; Talbert and Henikoff, 2017). In humans and mice, 11 different H1 variants have been identified (Fyodorov et al., 2018), while the *Arabidopsis* H1 family is formed by the H1.1, H1.2, and H1.3 histones (**Table 1**).

H1.1 and H1.2 —the replicative histones H1— are highly similar, whereas the H1.3 variant is shorter and lacks the (S/T)PXK motifs required for DNA binding (Kotliński et al., 2016). Consequently, the H1.3 variant has higher mobility within chromatin. H1.1 and H1.2 are enriched in heterochromatin, anti-correlate with gene expression (Rutowicz et al., 2015), and are also necessary for H3K27me3 deposition (Rutowicz et al., 2019). Alternatively, H1.3, although it is not abundant in the histone H1 pool, plays a specific role in the abiotic stress response. Under normal conditions, it is exclusively expressed in guard cells, but when the plant is exposed to a stimulus, such as light deficency, drought, and abscisic acid (ABA), H1.3 competes with H1.1 and H1.2 for the incorporation into the nucleosome (Rutowicz et al., 2015). Physiological and transcriptomic analyses of *h1.3* null mutants demonstrate that H1.3 is required for proper stomatal functioning under normal growth conditions and adaptive developmental responses to combined light and water deficiency (Rutowicz et al., 2015). The putative differences in the deposition patterns of H1.3 in different tissues in response to stress have not been explored.

## H2A and H2B families

The H2A histone family in *Arabidopsis* comprises four replicative H2A, four H2A.Z, three H2A.X, and two H2A.W (**Table 1**), composed of ~130 amino acid residues. H2A variants differ in the C-terminal motifs of their primary amino acid sequences (Kawashima et al., 2015). Some of these variants’ properties are conserved throughout the kingdoms. For instance, H2A.Z diverged from the canonical H2A early in eukaryotic evolution. H2A.Z properties have been thoroughly described in humans, mice, yeast, and plants. In all these kingdoms, H2A.Z histone is a replacement variant with similar roles in transcriptional regulation and DNA repair (Jarillo and Piñeiro, 2015; Giaimo et al., 2019; Gómez-Zambrano et al., 2019). In fact, H2A.Z sequences from different organisms show a higher similarity level than the H2A.Z and H2A within the same organism. The diverse relationship between H2A variants and gene expression explains histone variants’ impact on chromatin structure. H2A.X is distributed along the whole *Arabidopsis* genome, whilst H2A.W is enriched in pericentromeric regions, colocalizing with heterochromatin and transposable elements (TEs) (Lei and Berger, 2020; Bourguet et al., 2021). On the other hand, replicative H2A and H2A.Z are excluded from pericentromeric heterochromatin (Zilberman et al., 2008; Yelagandula et al., 2014). The exclusion of H2A.Z from pericentromeric heterochromatin has been linked to its shortened C-terminal tail, which is thought to limit the binding of the linker histone H1 to the core nucleosome particle (Osakabe et al., 2018).

Histone variants mediate the nucleosome adaptability to different stimuli. Changes in nucleosome composition directly reports on nucleosome stability (Osakabe et al., 2018). For instance, H2A.Z-H2B dimers are replaced more rapidly than H2A-H2B dimers (Brahma et al., 2017), conferring the genes covered by H2A.Z-H2B nucleosomes the ability to respond quickly to a stimulus. An intriguing plant H2A feature is that, in contrast to animals and yeast, H2A-containing nucleosomes are homotypic, since each variant associates only with itself (Osakabe et al., 2018).

The distribution of H2A.Z in the *Arabidopsis* genome is puzzling because of its dual, and perhaps interconvertible, deposition patterns. H2A.Z can be deposited either at the transcription start site (TSS) of a large set of constitutively expressed genes across cell types or at the gene-body of repressed genes (Coleman-Derr and Zilberman, 2012) associated with repressive H3K27me3. When incorporated at the TSS, it is thought to maintain genome integrity with stable transcription rates by facilitating the transcription of genes essential for plant survival (Mahrez et al., 2016). This process is thought to occur by reducing the energy required by the RNA polymerase II to overcome the first nucleosomal barrier (Sura et al., 2017). Over a decade ago, the involvement of the H2A.Z histone variant in gene responsiveness during environmental stress was elucidated by showing that H2A.Z is deposited within gene bodies in genes categorized as “hypervariable” (Coleman-Derr and Zilberman, 2012). Furthermore, transcriptome data of *h2a.z* knock-out mutant plants revealed a deregulation of *Arabidopsis* genes with high responsiveness scores, which correlates with those that have H2A.Z deposited on their gene body in the absence of stress. Notably, under normal conditions, gene-body H2A.Z deposition participates in the repression of genes involved in response to wounding, drought, ABA, salinity, UV light, heat, cold, immune response, defense response, and phosphate in *Arabidopsis* (Coleman-Derr and Zilberman, 2012). Since then, several authors have reported the implication of H2A.Z not only as a transcriptional regulator but also as a key player in gene repression under biotic and abiotic stress conditions (Cortijo et al., 2017; Sura et al., 2017; Nguyen and Cheong, 2018; Gómez-Zambrano et al., 2019; Bieluszewski et al., 2022).

The role of H2A.Z in stress resembles the function of the histone mark H3K27me3, as they both actively regulate the expression of hypervariable genes. Due to the similarities in the regulation of their targets, it was hypothesized that H2A.Z and H3K27me3 could functionally interact. In mouse embryonic stem cells, H2A.Z promotes chromatin compaction, favoring H3K27me3 deposition by the POLYCOMB REPRESSIVE COMPLEX 2 (PRC2) (Wang et al., 2018). Consistent with this, H3K27me3 is dependent on H2A.Z deposition in *Arabidopsis* (Dai et al., 2017; Carter et al., 2018). SWI2/SNF2-Related 1 Chromatin Remodeling Complex (SWR1), the complex incorporating the H2A.Z variant, is required for H3K27 trimethylation (Luo et al., 2020; Liu et al., 2021). However, the variant H2A.Z and the Polycomb modification H3K27me3 do not share most of their targets, as shown by the limited overlap of upregulated genes between *hta9-hta11*, defective in H2A.Z protein, and mutants of the Polycomb repressive complex 2 (PRC2) catalytic subunit *curly leaf (clf)* (Gómez-Zambrano et al., 2019). These differences suggest that the repression of targets *via* H2A.Z gene body-deposition targets a wide range of hypervariable genes and is not limited to stress-responsive genes. These data suggest an exciting timeframe in the repression of responsive genes, where deposition of H2A.Z by SWR1 is first needed to achieve PRC2 repression of hypervariable genes. H3K27me3 usually works in bivalent genes and is released from the environment of the gene it represses shortly after the stress stimulus (Zhang et al., 2011; Molitor et al., 2014; Zhang et al., 2020). ChIP-seq data of H2A.Z after stress are not available so far, and, consequently, it is not possible to conclude whether the H2AZ is evicted from the gene body or if it is deposited in a different region of the same locus —although it has been proposed that there is H2A.Z depletion from the gene body upon transcriptional activation (Sura et al., 2017). Establishing a timeline to clarify further the role of these critical actors in activating these repressed genes during the stress response remains unclear and needs further investigation.

The H2A.X variant, which only differs from the replicative H2A in the additional SQEF amino acid motif that H2A.X contains in its C-terminal tail, has been described to regulate the DNA damage response (DDR) (Dantuma and van Attikum, 2016; Lorković and Berger, 2017). In replicative stress, the ATR and ATM kinases phosphorylate H2A.X by a mechanism that is conserved in both animals and plants. The H2A.W.7 variant is necessary for DNA replication stress signaling in heterochromatin, which shows there might be an interaction between these H2A.X and H2A.W by the joint action of kinases to act in response to DNA damage in different regions of the *Arabidopsis* genome (Yelagandula et al., 2014; Lorković and Berger, 2017).

Compared with the extensive published studies defining H2A variants, only a handful of H2B variants have been characterized. Despite the similarities between H2A and H2B histones and the conserved status of their dimers, the *Arabidopsis* histone H2B family is formed by 11 genes that encode proteins of high sequence divergence (Jiang et al., 2020a). The expression of *Arabidopsis* H2B varies across development. Defining the role of H2B.8 —also known as H2B.S—is particular intriguing since this histone specifically accumulates during chromatin compaction of dry seed embryos (Jiang et al., 2020a; Buttress et al., 2022). The potential response of H2B proteins to abiotic stress has not been explored so far.

## H3 family

The *Arabidopsis* histone H3 family is one of the most studied and complex. It is composed of fifteen genes encoding nine H3 proteins with unique roles. The canonical form, the protein H3.1, is encoded by five intronless genes: *HTR1, HTR2, HTR3, HTR9*, and *HTR13.* This protein is only deposited during DNA replication and DNA repair. The histone H3.3, the best-characterized histone H3 variant, is encoded by the *HTR4, HTR5*, and *HTR8* genes and is incorporated throughout the whole cell cycle constitutively, in a DNA replication-independent manner, allowing a rapid chromatin adaptation to different environmental stimuli (March-Díaz and Reyes, 2009; reviewed in Talbert and Henikoff, 2017).

**Figure 2:**
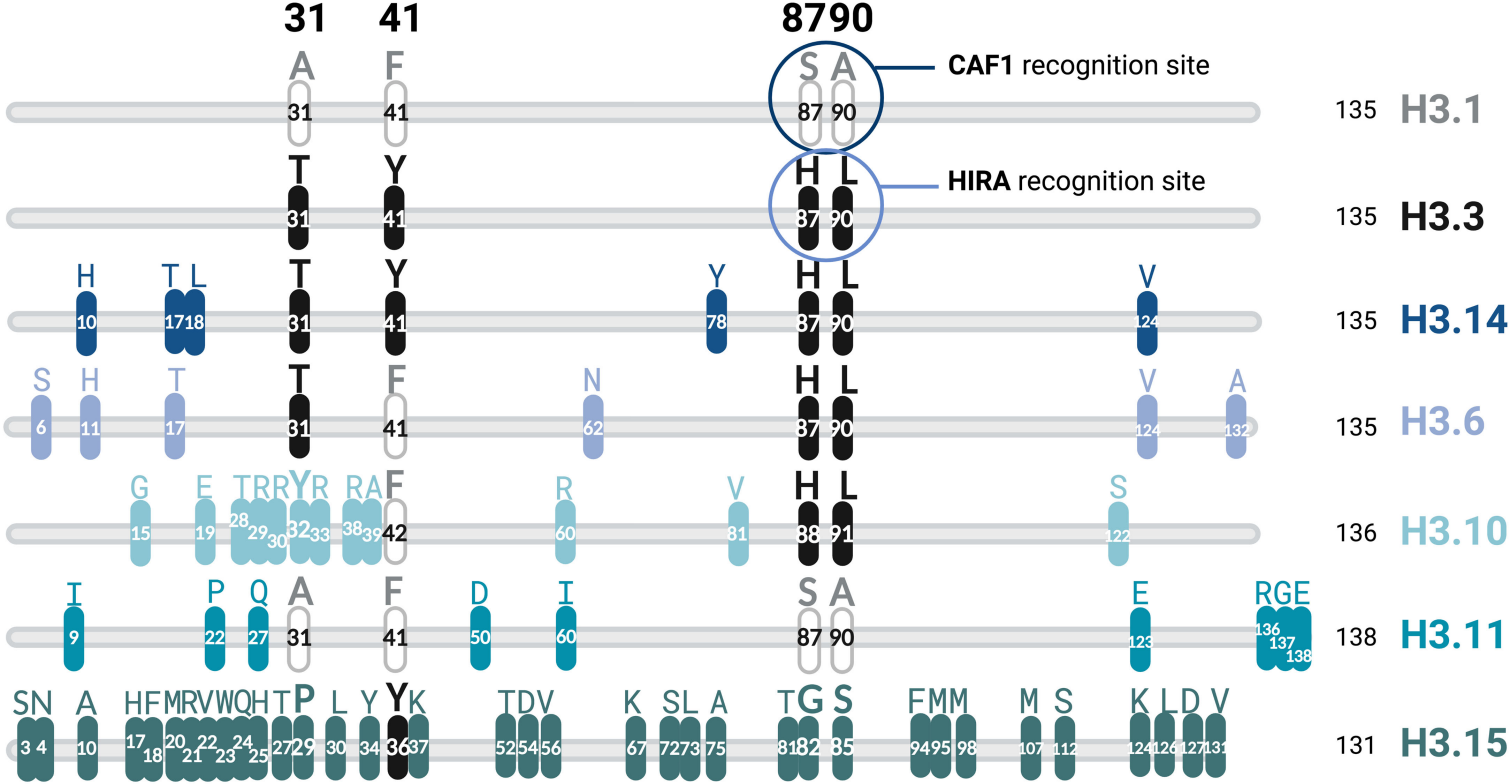
Differences in the amino acid sequence of the members of the histone H3 family in *Arabidopsis thaliana.* The length of each variant’s amino acid sequence is indicated in black.

The differences between the variants in the H1 and H2A histone families are driven by the distinct amino acid motifs, even domains, that they include in their sequence. Instead, the H3 family maintains a high amino acid homology degree. The differences in the H3 variants consist of changes of a relatively small number of aminoacids (**Figure 2**). H3.1 and H3.3 have unique properties, despite that their amino acid sequences differ only in 4 amino acid residues at positions 31, 41, 87, 90 (**Figure 2**). The substitution at position 41 of H3.1 is specific to dicotyledon plants (Lu et al., 2018). The differences in amino acid sequences between H3.1 and H3.3 are almost identical in plants and animals. Their distribution patterns are also highly similar across species (Ingouff and Berger, 2010; Stroud et al., 2012; Wollmann et al., 2012; Müller and Almouzni, 2014; reviewed in Loppin and Berger, 2020; Foroozani et al., 2022). This evidence of convergent evolution strongly points toward the importance of those specific residues in the function of the eukaryotic genome. Regarding histone H3 distribution along the *Arabidopsis* genome, ChIP-seq studies showed that H3.1 is enriched in heterochromatin, specifically in TEs and pericentromeric heterochromatin, colocalizing with histone modifications associated with gene repression such as H3K9me2, H3K27me1, H3K27me3 or DNA methylation, H2B ubiquitination, and RNA polymerase II occupancy (Stroud et al., 2012; Wollmann et al., 2012). In contrast, H3.3 is associated with active chromatin marks, including H3K4me3, H3K9me3, and H3K36me3. Therefore, H3.3 is associated with euchromatic regions, being deposited preferentially at the 3’ UTR end of constitutively expressed genes (Shi et al., 2011; Stroud et al., 2012; Wollmann et al., 2012; Shu et al., 2014).

Histone variants often play a role in the activation of certain groups of inducible genes. For example, H3.3 specifically regulates the expression of genes involved in environmental responses (Wollmann et al., 2017). Also, a recent study showed that H3.3 inhibits flowering by increasing the levels of H3K4me3 and H3K36me3 marks at the *FLOWERING LOCUS C (FLC)* gene (Zhao et al., 2021), although the specific mechanisms underlying the relationship between histone H3.3 and stress responses have not yet been clarified.

Genome architecture can be structurally shaped with the help of histone variants. A H3 variant known as CENH3 in plants —and CENP-A in mammals— is specifically incorporated in the centromere region (Malik and Henikoff, 2009; Fukagawa and Earnshaw, 2014; Müller and Almouzni, 2014). CENH3 is an essential protein that function in centromere organization and chromosome segregation (Ravi et al., 2010). The CENH3 amino acid sequence strongly diverges from that of the rest of H3 family members. A clear role of CENH3 in stress response has not been described. However, its expression was drastically reduced in the mutant background of *MUT9-LIKE KINASE1* and *2 (MLK1 and 2).* These kinases are in charge of H3.3 phosphorylation in a process that is dependent of the ABA pathway (Wang et al., 2015).

There is a group of atypical plant-specific H3 variants with specific substitutions in their N-terminal tail, encoded by the genes *HTR6* and *HTR14* that share features with both H3.1 and H3.3, although they are thought to be more similar to H3.3, as they contain the four critical amino acids (T31, Y41, H87, L90) in which H3.3 differ from H3.1 (**Figure 2**). Furthermore, H3.14 and H3.6 have been described to contain an enrichment of transcription factor binding sites implicated in salinity and drought stress responses in their respective promoter regions (Nunez-Vazquez et al., in preparation). Further differences are present in these atypical H3 variants, but the functional impact of these changes has yet to be explored. The atypical H3.15 has a distinguishing feature due to its lack of the K27 residue, which prevents the trimethylation of this residue by the Polycomb PRC2 complex and has been reported to be induced after wounding and has a role in cell fate reprogramming during plant regeneration (Yan et al., 2020). The sperm-specific H3.10 variant has an intricate role in heterochromatin formation and gene silencing, as it reprograms H3K27me3 during *Arabidopsis* spermatogenesis (Okada et al., 2005).

Several histone chaperones have been described to incorporate H3-H4 dimers in the nucleosome. CAF1 is the typical H3.1 chaperone, whereas HIRA commonly incorporates H3.3 by binding to its C-terminal tail’s H87 and L90 amino acids (Daniel Ricketts et al., 2015) (**Figure 2**). As many atypical histone variants (H3.6, H3.14, H3.10) contain the H87 and L90 residues, we hypothesize HIRA is likely to be responsible for their deposition, although further research is needed to demonstrate this assumption.

## Histone modifications

Chromatin stability is favored by the interaction of the negatively charged phosphate groups of DNA with the positively charged amino acids of histone proteins. The post-translational modifications (PTMs) of both histone tails and histone fold domains contribute to chromatin control and accessibility. The histone PTMs environment is founded and maintained by a set of highly coordinated enzymes (Kouzarides, 2007). PTMs are considered to favor the oscillation between relaxed or packaged chromatin configurations. However, whether histone PTMs are a cause or a consequence of changes in transcriptional regulation is a controversial and puzzling topic (Millán-Zambrano et al., 2022; Policarpi et al., 2022; Wang et al., 2022). On one hand, some evidence indicates that active histone modifications support transcription in an informative manner rather than serving as an essential regulatory function (Wang et al., 2022). On the other hand, a different study points towards *de novo* H3K4me3 deposition can induce major transcription activation (Policarpi et al., 2022). Here, we will discuss recent discoveries and summarize the current understanding of the regulation and function of histone post translational modifications in response to abiotic stress.

Histone PTMs include methylation, acetylation, phosphorylation, ubiquitination, and sumoylation, among others. These reactions are catalyzed by histone-modifying enzymes recruited to specific genomic regions (Kouzarides, 2007). The chromatin landscape of active genes is preferentially associated with highly acetylated histones, whereas inactive genes are associated with hypoacetylated histones (Hebbes et al., 1988). The general assumption is that acetylation of lysine and arginine residues reduces the DNA-histone interactions and relaxes the chromatin structure, resulting in increasing accessibility to the DNA of the transcriptional machinery (Allis and Jenuwein, 2016). The association between histones and DNA is also regulated by histone methylation. Due to the neutral character of this modification, methylation of amino acids does not directly perturb nucleosome stability (Xiao et al., 2016), although it affects the local hydrophobicity. Hence, it appears in association with actively transcribed or repressed genes, depending on the methylated amino acid residue (Xiao et al., 2016; Yung et al., 2021). In contrast, the phosphorylation of threonine, serine, and tyrosine adds an extra negative charge to the chain, weakening the DNA-histone interaction. Ubiquitination of lysines, consisting of the addition of small amino acid chains to the histone tail, also compromises nucleosome stability. H2Aub has been associated with gene silencing, whereas H2Bub is linked to transcriptional activation. The specific mechanism of transcription regulation by ubiquitination has not yet been clarified (Zhou et al., 2017; Zhou et al., 2018).

Although nucleosomes are present in all eukaryotic cells, the role of specific PTMs varies between animals and plants. For example, H3K9me3, a constitutive heterochromatin mark in mammals, is present in plant euchromatin, whereas the dimethylated state, H3K9me2, is associated with plant heterochromatin (Lippman et al., 2004; Zhang et al., 2008). The monomethylation of H3K27 is a plant-specific heterochromatin mark, although it also appears at lower level in repressed genes of euchromatin (Jacob et al., 2009). H3K27me3 regulates facultative heterochromatin —specific regions of the genome that behave as heterochromatin in some cells or developmental stages but as euchromatin in others— both in plants and animals (Schuettengruber et al., 2007; Zheng and Chen, 2011; Makarevitch et al., 2013), as it is the case for H3K4me3 in actively transcribed genes (Zhang et al., 2009).

**Figure 3:**
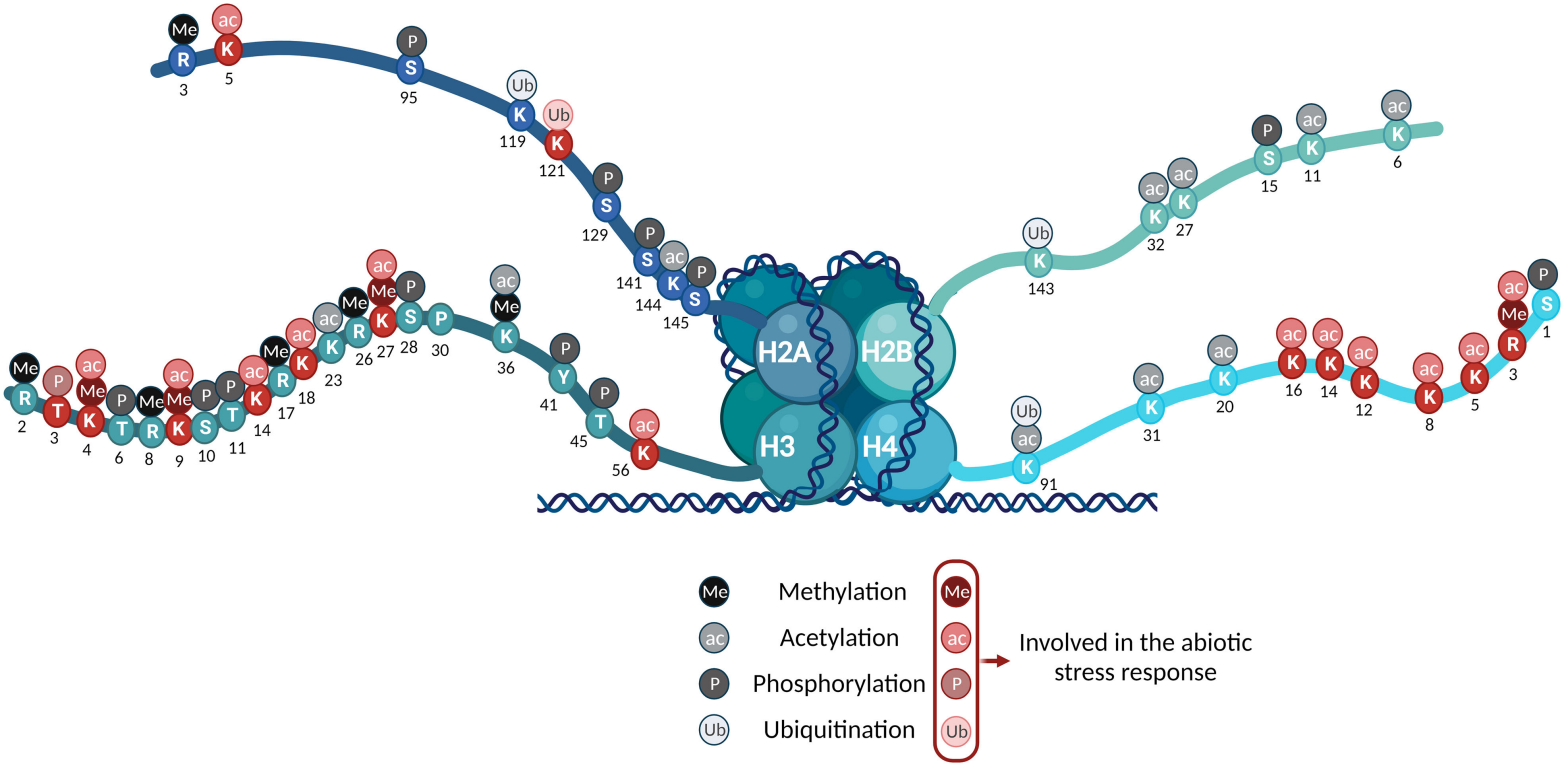
Summary of the histone PTMs in the nucleosome’s core histones in *Arabidopsis*. The PTMs detected are color-coded as indicated. Those involved in the abiotic stress response are highlighted in red.

Several studies have reported that PTMs are involved in seed formation, flowering, and biotic and abiotic stress responses (Cao et al., 2008; Zou and Mallampalli, 2014; Huang et al., 2016; Zhou and Zeng, 2017). In the presence of stress, the plant needs to reorganize and optimize its resources. (Atkinson and Urwin, 2012). For that purpose, it pauses different ongoing processes, such as protein translation and cell elongation, and prioritizes those that are strictly necessary for plant survival (Muñoz and Castellano, 2012; Yamamoto, 2019). Modifying the local chromatin landscape during the stress response does not comprise a specific PTM. Instead, it involves globally induced changes that could be summed up as (1) an increase in histone acetylation in the promoters and gene bodies of drought-inducible genes and (2) derepression of hyperresponsive targets by histone and DNA demethylation (To and Kim, 2014) (**Figures 1**, **3**).

The induction of the abiotic stress-responsive genes is independent of the mechanism of the stress-memory (Ding et al., 2012). Consequently, we consider that the regulation of stress memory is out of the scope of this review article. We have selected a list of recent review articles that detail the stress memory process (Oberkofler et al., 2021; Liu et al., 2022; Perrella et al., 2022).

## Histones acetyltransferases

Histone acetyltransferases (HATs) catalyze the transfer of the acetyl group from acetyl-CoA to the amino group of the lysine residues at the N-terminal tail of the histones. This reaction results in an acetylated lysine that compromise the interaction of the histone with the negatively charged DNA, leading to an open status of the chromatin (Smith and Denu, 2009). In presence of diverse abiotic agents —heat, salt, limited water availability— there is a global increase in histone acetylation (Pandey et al., 2002; Earley et al., 2007), (**Figure 1**). Acetylation marks allow the binding of stress-specific transcription factors —such as ABRE or DREB— during the stress response to areas of the genome that are generally silent (Kim et al., 2014; Widiez et al., 2014).

In *Arabidopsis*, 12 different HATs belong to four families: the GNAT/HAG, the MYST/HAM, the p300/CBP/HAC and the TAFII250/HAF families (Pandey et al., 2002; Fina et al., 2017) (**Table 2**). They regulate plant development, flowering time, and some specific processes of abiotic stress response that include response to light, salt tolerance, DNA damage and hormonal pathways (Earley et al., 2007; Xu et al., 2012; Xiao et al., 2013).

**Table 2:** Classification of histone acetyltransferases in *Arabidopsis*.

Enzyme group	Family	Regulator	Gene	Target	Role in Stress (References)
Acetyltransferases	GNAT	HAG1	*At3G54610*	H3K14	Salt tolerance (Zheng et al., 2019). Cold and heat stress (Hu et al., 2015)
		HAG2	*At5G56740*	H4K12	(Pavangadkar et al., 2010)
		HAG3	*At5G50320*	H3K56 and H4K5	UVB light response (Fina et al, 2017)
	MYST	HAG4/HAM1	*At5G64610*	H4K5	ABA, UVB light responses, DNA damage repair (Campi et al., 2012; Umezawa et al., 2013)
		HAG5/HAM2	*At5G09740*	H4K5	ABA, UVB light responses, DNA damage repair (Campi et al., 2012; Umezawa et al., 2013)
	CBP	HAC1	*At1G79000*	H4K14, H3K9	Ethylene response (Li et al., 2014). Heat (Roca Paixão et al., 2019)
		HAC2	*At1G67220*		
		HAC4	*At1G55970*		
		HAC5	*At3G12980*	H3K9	Ethylene response (Li et al., 2014)
		HAC12	*At1G16710*	H3K9	
	TAF11250	HAF1	*At1G32750*	H3Ac, H4Ac	
		HAF2	*At3G19040*	H3Ac, H4Ac	

The GNAT superfamily member histone acetyltransferases GENERAL CONTROL NONDEREPRESSIBLE 5 (GCN5), encoded by *HAG1*, has been positively linked to cold and heat stress (Pavangadkar et al., 2010; Hu et al., 2015) and with the positive regulation of salt tolerance (Zheng et al., 2019). GCN5 was the first HAT identified in *Arabidopsis*. Transcriptomic analyses of *gcn5* mutant show pleiotropic defects in the expression of genes involved in plant development and adaptation to environmental conditions (Cohen et al., 2009; Servet et al., 2010; Kim et al., 2018; Wang et al., 2019; Zheng et al., 2019). Importantly, under salt stress, *gcn5* plants present inhibited growth compared to wild type plants (Zheng et al., 2019). The preferential GCN5 acetylation sites are the lysine residues of histone H2B and H3, with a lower preference for histone H4 (Fan et al., 2017; Mutlu and Puigserver, 2020). In fact, a decrease in the H3K9ac and H3K14ac marks has been reported in *gcn5* mutants under salt stress (Li et al., 2022).

HAC1 and HAC5, two members of the p300/CBP family, participate in the ethylene response. The transcriptional levels of the ethylene response factors (ERFs) *ERF1, ERF4, ERF6* and *ERF11* significantly increase in the *hac1hac5* double mutant (Li et al., 2014). It is possible that HAC1 and HAC5 might as well be involved in salinity stress response, as there is a close relationship between ethylene and salinity tolerance (Tao et al., 2015). Nevertheless, further research is needed to demonstrate it.

The *Arabidopsis* MYST family includes homologs of the catalytic subunit of the Nucleosome Acetyltransferase of the yeast H4 (NuA4) complex. Its components, HAG4/HAM1 and HAG5/HAM2, regulate general developmental processes in the plant, such as flowering, gametogenesis, chlorophyll synthesis, cell growth, and ploidy (Latrasse et al., 2008; Zacharaki et al., 2012; Crevillén et al., 2019). HAG4/HAM1 and HAG5/HAM2 also take part in ABA and UVB light responses, and other cell functions such as transcriptional activation and DNA damage repair (Campi et al., 2012; Umezawa et al., 2013).

## Histone deacetylases

The opposite action of HATs is conducted by histone deacetylases (HDAC). These enzymes catalyze the hydrolysis of the acetyl group from the amine of acetyl-lysine residues within histone tails. The 16 HDACs encoded in the *Arabidopsis* genome (**Table 3**) are organized into three families (RPD3/HDA1, HD2, and SIR2).

**Table 3:** Classification of histone deacetylases in *Arabidopsis*.

Enzyme group	Family	Regulator	Gene	Target	Role in Stress (References)
Deacetylases	RPD3/HDA1	HDA2	*At5G26040*	H3Ac and H2BAc	
		HDA5	*At5G61060*	H3Ac	
		HDA6	*At5G63110*	H3K9, H3K14, H4	Drought and salt stress (Chen and Wu, 2010; Kim et al., 2017), cold (To et al., 2011; Jung et al., 2013; Luo et al., 2017), heat (Popova et al., 2013), pathogen defense, JA, and salicylic acid-mediated defense responses (Zhou et al., 2005; Wu et al., 2008; Choi et al., 2012)
		HDA7	*At5G35600*	H3K9, H3K14	
		HDA8	*At1G08460*		
		HDA9	*At3G44680*	H3K9	Drought and salinity (Mehdi et al., 2016; Zheng et al., 2016)
		HDA10	*At3G44660*		
		HDA14	*At4G33470*		
		HDA15	*At3G18520*		Drought (Lee and Seo, 2018; Tu et al., 2022)
		HDA17	*At3G44490*		
		HDA18	*At5G61070*		
		HDA19	*At4G38130*	H3K9, H3K14, H3K18, H2B	ABA and salt stress (Chen and Wu, 2010). Pathogen defense, JA, and salicylic acid-mediated defense responses (Zhou et al., 2005; Wu et al., 2008; Choi et al., 2012)
	HD2	HDT1/HD2A	*At3G44750*	H3K18, H3K27, andH2B	Repressed in ABA and salt (Luo et al., 2012)
		HDT2/HD2B	*At5G22650*	H3K18 and H3K27	Repressed in ABA and salt (Luo et al., 2012)
		HDT3/HD2C	*At5G03740*	H3K9, H3K18	Salinity and drought tolerance (Luo et al., 2012), cold (Park et al., 2018), heat (Buszewicz et al., 2016)
		HDT4/HD2D	*At2G27840*	H3K27	Salinity tolerance, cold and drought (Luo et al., 2012; Han et al., 2016)
	SIR2	SRT1	*At5G55760*	H3K9	Ethylene response (Zhang et al., 2018)
		SRT2	*At5G09230*	H3K9	Ethylene response (Zhang et al., 2018)

In *Arabidopsis*, HDA6 and HDA19 are the most extensively studied HDACs (Mehdi et al., 2016). They belong to the RPD3/HDA1 family. HDA6 and HDA19 have similar developmental functions. Both participate in pathogen defense systems, JA, and salicylic acid-mediated defense responses (Zhou et al., 2005; Wu et al., 2008; Choi et al., 2012), regulation of flowering, senescence (Wu et al., 2008; Yu et al., 2011; Mehdi et al., 2016), and abiotic stress responses (Chen and Wu, 2010). The fundamental difference between HDA6 and HDA19 is the antagonistic function, positive and negative, respectively, they have in the regulation of salt stress (reviewed in Luo et al., 2017).

HDA19 represses gene expression upon ABA and drought treatments by four different ways: 1) interaction with the ethylene response factor ERF7 and the transcriptional repressor SIN3, originating a repressive complex that silences stress-responsive genes (Song et al., 2005); 2) binding to SIN3-LIKE1 (SNL1) and SIN3-LIKE2 (SNL2), homologs of SIN3, to form a repressive complex that prevents ABA biosynthesis *via* the deacetylation of H3K9/14/18 (Wang et al., 2013); 3) formation of a complex with MSI1 that represses expression of genes in the ABA pathway such as the ABA receptors PYL4, PYL5, and PYL6 (Mehdi et al., 2016); and 4) binding to HDA6 and HDC1 and deacetyl K3K9/K14 in response to drought stress (Perrella et al., 2013).

HDA6 is upregulated by cold stress. This enzyme regulates cold-responsive (COR) genes during freezing tolerance (Park et al., 2018). HDA6 forms complexes with MSI4 and MSI5, that cause histone deacetylation in specific target loci, leading to transcriptional gene silencing (Gu et al., 2011; Mehdi et al., 2016).

The histone deacetylase HDA9, another member of the RPD3/HDA1 family, is a negative regulator of the ABA pathway. *hda9* loss-of-function mutant displays increased tolerance to dehydration and upregulation of drought-responsive genes. During drought conditions, HDA9 interacts with critical components of the ABA pathway, such as ABI4 (Baek et al., 2020), and results in the induction of critical enzymes in the ABA catabolic pathways like ABA 8’-hydroxylases, encoded by CYP707A1 and CYP707A2 (Baek et al., 2020). HDA9 is also particularly important because it collaborates with the PRC2 complex by deacetylating H3K27 prior to its trimethylation (Qian et al., 2012).

HDA15 participates in the regulation of several warm temperature genes, including *HEAT SHOCK PROTEIN 20 (HSP20), INDOLE-3-ACETIC ACID INDUCIBLE 19 (IAA19)*, and *IAA29* (Shen et al., 2019). HDA15 participates in the ABA pathway. On one hand, it interacts with the transcription factor MYB96 to repress the expression of *RHO GTPASE OF PLANTS* in response to ABA (Lee and Seo, 2019). On the other hand, HDA15 interacts with MAC3A and MAC3B, subunits of the MAC complex, by a process enhanced by ABA. Moreover, *hda15* and *mac3a/mac3b* mutants are ABA insensitive in seed germination and hyposensitive to salinity (Tu et al., 2022).

The expression of HD2A, HD2B, HD2C, and HD2D —members of the HD2A deacetylase family— is repressed by ABA and NaCl, which indicates their potential role in stress response. Overexpression of HD2D and HD2C results in increased drought tolerance (Sridha and Wu, 2006; Han et al., 2016). The expression of the ABA-responsive genes *ABI1* and *ABI2* increase in *hda6, hd2c*, and *hda6/hd2c-1* mutant backgrounds, which was associated with increased histone H3K9/K14 acetylation (Luo et al., 2012). In the regulation of salinity tolerance, HD2C, together with HDA6 and HD2D, act as positive regulators (Luo et al., 2012).

In summary, HDA9 and HDA19 negatively regulate salt stress tolerance (Mehdi et al., 2016; Zheng et al., 2016; Ueda et al., 2017), while HDA6, HD2C, and HD2D positively regulate salinity tolerance (Chen and Wu, 2010; Chen et al., 2010; Luo et al., 2012; Han et al., 2016). These roles are further supported by the phenotypes of the previously mentioned HDAC mutants (reviewed in Ueda and Seki, 2020).

## Histone methyltransferases

Histone methyltransferases catalyze mono-, di- and trimethylation of the amino group of lysines and arginines. This process is dependent on S-adenosyl-L-methionine (Smith and Denu, 2009). In plants, there are only eight histone lysine methylation sites: H3K4, H3K9, H3K26, H3K27, H3K36, H3K79, H4K20 and H1K26, and six arginine methylation sites: H3R2, H3R8, H3R17, H3R26, H4R3 and H4R17 (Zhang and Reinberg, 2001; Springer et al., 2003; Liu et al., 2010; Pontvianne et al., 2010; reviewed in Ueda and Seki, 2020).

The *Arabidopsis* genome contains 49 genes encoding SET domain-containing (SDG) methyltransferases (Baumbusch et al., 2001; Ng et al., 2007). Out of the 49 SDG proteins, 31 have histone lysine methyltransferase (HKMT) activity and are divided into five classes (I to V) based on their domain architectures (**Table 4**) (Baumbusch et al., 2001; Springer et al., 2003; Ng et al., 2007). In addition, there is an additional HKMT family, known as telomeric silencing 1-like (DOT1), which does not contain a SET domain and specifically adds methyl groups at the telomeric regions of H3K79 (Ng et al., 2002). Protein arginine methyltransferases (PRMTs) are classified as Type I or Type II, depending on the position of the methyl group on the guanidine of the methylated arginine (Hernando et al., 2015).

**Table 4:** Classification of histone methyltransferases in *Arabidopsis*.

Enzyme group	Family	Regulator	Gene	Target	Role in Stress (References)
Lysine Methyltransferases	I, E(Z)	CLF/SDG1	*At2G23380*	H3K27	
		SWN/SDG10	*At4G02020*	H3K27	
		MEA/SDG5	*At1G02580*	H3K27	
	II, ASH1	ASHH1/SDG26	*At1G76710*	H3K36	
		ASHH2/SDG8	*At1G77300*	H3K36	Immunity defense (Lee et al., 2016)
		ASHH3/SDG7	*At2G44150*	H3K36	
		ASHH4/SDG24	*At5G59960*	H3K36	
		ASHR3/SDG4	*At4G30860*	H3K36/H3K4	
	III, TRX	ATX1/SDG27	*At2G31650*	H3K4	Dehydration and osmotic stress (Ding et al., 2011). Heat (Song et al., 2021)
		ATX2/SDG30	*At1G05830*	H3K4	
		ATX3/SDG14	*At3G61740*	H3K4	
		ATX4/SDG16	*At4G27910*	H3K4	Drought (Liu et al., 2018)
		ATX5/SDG29	*At5G53430*	H3K4	Drought (Liu et al., 2018)
		ATXR3/SDG2	*At4G15180*	H3K4	
		ATXR7/SDG25	*At5G42400*	H3K4	Heat (Song et al., 2021); Immunity defense (Lee et al., 2016)
	IV, SET+PHD	ATXR5/SDG15	*At5G09790*	H3K27	
		ATXR6/SDG34	*At5G24330*	H3K27	
	V, SU(VAR)	SUVH1/SDG32	*At5G04940*	H3K4, H3K9	
		SUVH2/SDG3	*At2G33290*	H3K9, H3K27, H4K20	
		SUVH3/SDG19	*At1G73100*	H3K9	
		SUVH4/SDG33	*At5G13960*	H3K9	
		SUVH5/SDG9	*At2G35160*	H3K9	
		SUVH6/SDG23	*At2G22740*	H3K9	
		SUVH7/SDG17	*At1G17770*	H3K9	
		SUVH8/SDG21	*At2G24740*	H3K9	
		SUVH9/SDG22	*At4G13460*	H3K9	
		SUVR1/SDG13	*At1G04050*	H3K9	
		SUVR2/SDG18	*At5G43990*	H3K9	
		SUVR3/SDG20	*At3G03750*	H3K9	
		SUVR4/SDG31	*At3G04380*	H3K9	
		SUVR5/SDG6	*At2G23740*	H3K9	
	DOT1	DOT1	*At2G36120*	H3K79	
Arginine Methyltransferases	PRMT	PRMT1	*At2G19670*	H4R3	
		PRMT3	*At3G12270*	H4R3	
		PRMT4A	*At5G49020*	H3R2, H3R17	
		PRMT4B	*At3G06930*	H3R2, H3R17	
		PRMT5/CAU1/SKB1	*At4G31120*	H4R3	Drought (Fu et al., 2013) and salinity (Zhang et al., 2011)
		PRMT6	*At3G20020*	H3R2	
		PRMT7	*At4G16570*	H4R3	
		PRMT10	*At1G04870*	H4R3	
		PRMT11	*At4G29510*		

Plant SET proteins are classified into five classes: E(Z), ASH1, TRX (trithorax), PHD and SU(VAR) (**Table 4**).

The most common forms of methylation consist of the trimethylation of H3K27 and H3K4. H3K27me3 increases chromatin condensation and limits the recruitment of the transcriptional machinery and transcription factors to genes. Thus, H3K27me3 is associated with gene repression (Aranda et al., 2015; Zhao et al., 2021). In contrast, H3K4me3 colocalizes with actively transcribed genes, where it promotes the recruitment of transcription initiation factors to promoters of target genes (Lauberth et al., 2013; Zhao et al., 2021).

The leading writers of H3K27me3 in plants and animals are the PRC2 complexes. In *Arabidopsis*, the histone methyl transferase of the PRC2 complex and EZ homologs are *MEDEA (MEA)*, *CURLY LEAF (CLF)* and *SWINGER (SWN)* (Makarevitch et al., 2013). The H3K27me3 mark has an intricate relationship with stress. It is a mark of facultative heterochromatin, involved primarily in the repression of developmentally regulated genes (Füßl et al., 2018). The H3K27me3 mark gives a more plastic structure to chromatin than constitutive heterochromatin. This structure allows condensation or decondensation of regions and permits transcription in temporal and spatial contexts, such as the derepression of genes involved in the abiotic stress response. Together with H3K4me3, it can have an implication on bivalent and responsive genes (Zhao et al., 2021).

The members of the trithorax family are responsible of H3K4 trimethylation. ATX1 drives H3K4me3 methylation in response to drought and osmotic stress (Ding et al., 2011). ATX1 together with ATRX7 regulate the expression of heat stress-responsive genes, not only during heat stress but also during stress recovery (Song et al., 2021). *atx4* and *atx5* mutants, also members of the trithorax family, showed increased tolerance to drought and salt stresses (Liu et al., 2018).

The loss of function of CAU1/PRMT5/SKB1, a member of the Type II PRMT family, results in salt hypersensitivity (Zhang et al., 2011). This enzyme catalyzes the addition of methyl groups to H4R3. In the presence of the salt stimulus, SKB1 dissociates from chromatin, leading to demethylation of arginine residues, and the transcription of stress-responsive genes (Fu et al., 2013).

## Histone demethylases

Histone demethylases perform the antagonistic reaction to histone methyltransferases. It consists of the removal of the methyl group of the lysines and arginine residues of H3 and H4 tails. The nature of histone demetylation is intriguing due to the irreversible nature of the C-N bond. The first histone demethylase activity was identified in 1973 (Paik and Kim, 1973). There are 25 histone demethylase genes encoded in the *Arabidopsis* genome (**Table 5**) organized into two families: the FAD-dependent LSD/LDL/FLD and Jumonji C JMJ (Tsukada et al., 2006; Gu et al., 2016).

**Table 5:** Classification of histone demethylases in *Arabidopsis*.

Enzyme group	Family	Regulator	Gene	Target	Role in Stress (References)
Demethylases	LSD/LDL/FDL	LDL1	*At1G62830*	H3K4me1/2	
		LDL2	*At3G13682*	H3K4me1/2	
		LDL3	*At4G16310*	H3K4me2	
		FDL	*At3G10390*	H3K4me1/2	
	KDM4/JHDM3	JMJ11/ELF6	*At5G04240*	H3K27me2/3, H3K9me3	
		JMJ12/REF6	*At3G48430*	H3K27me2/3, H3K9me3, H3K4me2/3, H3K36me2/3	
		JMJ13	*At5G46910*	H3K27me3	
	KDM5/JARID1	JMJ14	*At4G20400*	H3K4me1/2/3	High temperatura (HT) (Cui et al., 2021)
		JMJ15	*At2G34880*	H3K4me1/2/3	Salinity (Shen et al., 2014) HT (Cui et al., 2021)
		JMJ16	*At1G08620*	H3K4me3	
		JMJ17	*At1G63490*	H3K4me1/2/3	Dehydration (Huang et al., 2019)
		JMJ18	*At1G30810*	H3K4me2/3	
		JMJ19	*At2G38950*	H3K4me3	
		JMJ21	*At1G78280*	H3K4me3	
		JMJ22	*At5G06550*	H3R2me2, H4R3me1/2	
	KDM3/JHDM2	JMJ24	*At1G09060*	H3K9	
		JMJ25	*At3G07610*	H3K9me1/2	
		JMJ26	*At1G11950*	H3K9me2	
		JMJ27	*At4G00990*	H3K9me2	ABA and drought (Wang et al., 2021)
		JMJ28	*At4G21430*	H3K9me2	
		JMJ29	*At1G62310*	H3K9me2	
	JmjC domain-only	JMJ20	*At5G63080*	H3R2me2, H4R3me1/2	
		JMJ30	*At3G20810*	H3K27me2/3, H3K36me2/3	
		JMJ31	*At5G19840*		
		JMJ32	*At3G45880*	H3K27me3	

LSD1 was the first isolated demethylase (David Allis et al., 1980). This enzyme catalyzes the reduction of FAD to FADH2 and oxidizes the methylated lysine, resulting in an imine intermediate (Smith and Denu, 2009). The mechanism of histone demethylation by LSD1 is highly conserved among most eukaryotes (Lan et al., 2007b; Lan et al., 2007a; Liu et al., 2007; Opel et al., 2007; Rudolph et al., 2007; Katz et al., 2009). Another potential mechanism is the conversion of methylarginine to citrulline by a peptidyl arginine deiminase (Wang et al., 2004).

The histone demethylases included in the JMJ family contain a JmjC domain, which catalyzes the histone demethylation through the oxidation of ferrous ions Fe (II) and α- ketoglutarate (α-kg) (Lu et al., 2010). JMJ15 and JMJ17 demethylases take part in salinity and dehydration stress response, respectively (Liu et al., 2010; Shen et al., 2014; Xiao et al., 2016; Huang et al., 2019). There is an accumulation of lignin in the *jmj15* mutant, although the regulation of lignin biosynthetic genes by JMJ15 still remains uncertain. It would be interesting to study whether JMJ15 and the HAT GCN5 participate in a common regulatory pathway in cell wall modification (Yung et al., 2021). *jmj15* exhibits increased sensitivity to salinity stress. Similarly, overexpression of JMJ15 increases salinity tolerance in the plant and enhance seed germination under salt treatment (Yang et al., 2012; Shen et al., 2014). The loss-of-function mutants of JMJ17 display dehydration stress tolerance and ABA hypersensitivity regarding stomatal closure. During high temperature conditions, JMJ14 and JMJ15 remove H3K4me3 from transcriptional repressors of responsive genes in response to trigger thermomorphogenesis (Cui et al., 2021).

A recent study details the role in abiotic stress of JMJ27 (Wang et al., 2021). They revealed that JMJ27 positively regulates both ABA and drought-responsive genes and establishes a permissive chromatin state to enable an efficient transcriptional induction upon drought stress conditions (Chen et al., 2010; van Dijk et al., 2010). This is achieved by the demethylation of H3K9me2. Likewise, JMJ27 may function together with drought stress-activated H3K4 methyltransferase and histone acetyltransferase to co-activate their target genes under drought stress conditions.

## Histone ubiquitination

Although acetylation and methylation are the most studied PTMs, there are additional modifications that influence chromatin accessibility. Histone ubiquitination comprises the incorporation of a 76-amino acid polypeptide into lysine residues of histones. This modification occurs mainly in H2A and H2B histones and is catalyzed by the formation of an isopeptide bond between the carboxy-terminal glycine of ubiquitin and the ϵ-group of a lysine residue on the carboxy-terminal tail of the histones. Substrates can be both poly- and monoubiquitinated. Polyubiquitination creates an irreversible signal for proteasomal-mediated degradation, whereas monoubiquitination generates a regulatory signal, which can be reversed by the action of ubiquitin-specific proteases (USPs/UBPs) called deubiquitinating enzymes (DUBs) (Zhou et al., 2017).

*Arabidopsis* E3 ubiquitin ligases (HUB1 and HUB2) and E2 ubiquitin conjugases (UBC1 and UBC2) are responsible for histone H2B mono-ubiquitination (H2Bub) (Cao et al., 2008). H2Aub is preferentially linked to transcriptional repression by counteracting H3K4me3. Specifically, the PRC1 complex catalyzes the monoubiquitination of H2A.ZK129 in a process linked to transcriptional repression (Gómez-Zambrano et al., 2019). On the other hand, H2Bub is a significant regulator of transcriptional activation, as it is required for H3K4me3 and H3K36me3. The monoubiquitination of H2B leads to the activation of responsive genes involved in abiotic and biotic stress that includes drought, salt, fungal pathogens, cold, heat and immune responses (Cao et al., 2008; Dhawan et al., 2009; Zou et al., 2014; Zhou and Zeng, 2017; Chen et al., 2019; Sun et al., 2020).

## Histone phosphorylation

Histone phosphorylation consists of the addition of a phosphate group, and thus, of a negative charge, to serine, threonine, or tyrosine residues of the N-terminal tail of histones (**Figure 3**). This modification is involved in response to DNA damage, extracellular signals, and mitosis, where it leads to chromatin condensation in prophase (Dai et al., 2005; Houben et al., 2007; Rossetto et al., 2012; Wang and Higgins, 2013; Wang et al., 2015). The phosphorylation process is conserved along eukaryotes (Bi et al., 2011; Pirrotta, 2015).

There is also induction of H3 phosphorylation in response to abiotic stress, although the specific molecular mechanisms of the response are not clearly understood. H3S10ph is encompassed with acetylation in response to salt stress and cold (Sokol et al., 2007), suggesting that H3 phosphorylation is associated with transcription reprogramming after stress inducement. The phosphorylation of H3T3ph increases in pericentromeric regions after drought stress treatments (Wang et al., 2015) and is thought to be important in the maintenance of heterochromatin, suggesting that phosphorylation is implicated in gene silencing upon abiotic stress.

## Conclusions and future perspectives

How chromatin marks affect transcription is a hot topic that brings the attention of epigenetics researchers from diverse backgrounds and fields. Thus, whether chromatin changes cause or correlate with the changes in gene expression is an area of active debate (Millán-Zambrano et al., 2022; Policarpi et al., 2022; Wang et al., 2022). Over the last decade, different studies have addressed this question with different technologies including ChIP-seq, CUT&RUN, CUT&Tag and TADs (Barski et al., 2007; Lieberman-Aiden et al., 2009; Dixon et al., 2012; Brind’Amour et al., 2015; Skene and Henikoff, 2017; Hainer and Fazzio, 2019; Kaya-Okur et al., 2019; Deng et al., 2022). On one hand, some evidence support that the marks are not instructing the activation/silencing, but instead they are informative of DNA processes (Wang et al., 2022). On the other hand, recent data support that some histone PTMs, such as H3K4me3, directly drive changes in gene expression (Policarpi et al., 2022). We consider that the answer to this question is far from being simple. It is likely that some histone PTMs may cause the initiation of DNA processes, such as transcriptional activation. In these cases, the histone PTM could be responsible for driving the genomic response. Similarly, there is a good chance that other histone PTMs are written as a consequence of these processes. For example, as the trace of a polymerase in a specific region of the genome. The mechanism that determines which marks acts as instructors or consequences of a genomic response depends on the context provided by the tissue, the developmental stage and the genomic landscape.

This topic is particularly intriguing during abiotic stress conditions, where the timing and order of the histone modifications is a crucial step to decipher the mechanism guiding the stress response. For that purpose, it is essential to establish a timeline of the epigenetic reprogramming in this scenario. During the early response, which ranges from the first to the fifth hour, a signaling network drives the binding of stress-specific transcription factors —DREB, ABRE— and the transcriptional machinery (Geng et al., 2013). Deciphering which epigenetic mark or histone variant is deposited/removed first after the abiotic stress would be the basis to understand better the intricate relationship between the histone variants and modifications. New studies focused on the temporal analysis of loss of and gain of function mutants of the enzymes that drive the major epigenetic regulators (H3K27me3, H3K4me3 and H2A.Z) will be crucial to establish the temporal epigenetic dynamics.

Bivalent chromatin is composed of epigenetic marks that play opposite roles on gene expression and co-localize in the same genomic regions (Voigt et al., 2013; Zhao et al., 2021). The H3K27me3/H3K4me3 pair of marks is the most usual form of bivalent chromatin. The first bivalent genes identified participate in cell differentiation in human embryonic stem cells (Bernstein et al., 2006). Since then, bivalent genes were identified in distinct species. An example of bivalent gene in *Arabidopsis* is the *FLOWERING LOCUS C (FLC)* (Jiang et al., 2008). The main hypothesis is that bivalent chromatin serves as a fast mechanism inducing developmentally regulated genes during differentiation (Bernstein et al., 2006). As the early stress response requires a rapid activation of responsive genes, a hypothesis is that poising of genes for transcriptional activation could be a mechanism for a fast gene regulation in response to abiotic stress. However, the role of bivalency marks has not been properly characterized in whole organisms nor during stress responses —with the exception of cold stress in potato tuber (Zeng et al., 2019)— so we consider it is an interesting topic of research.

There are histone variants that behave differently depending on the tissue they are deposited. If we take the chromatin organization within the sperm cells as an example, we find a regulatory network of histone PTMs and variants that define the accessibility to the transcriptional machinery. Histone variants H3.10 and H2B.8 are specific of sperm cells and represent the major pool of histones H3 and H2B, respectively (Okada et al., 2005; Jiang et al., 2020a; Buttress et al., 2022). Additionally, H3K27 is demethylated in sperm nuclei in a well-coordinated system in which the loss of H3K27me3 is associated to an increase of H3K4me3 in those genes required for embryo patterning, seed dormancy and flowering (Borg et al., 2020). Altogether, this suggest there is an intricate and well-conserved relationship between histone variants and modifications in specific tissues during development. Therefore, the tissue specificity of some epigenetic players rise the question of whether there are tissue-specific epigenetic changes during the abiotic stress response. Due to the nature of the stress response, it is possible that external organs and tissues reprogram their epigenetic landscape differently from internal tissues.

There is a need to perform studies that not only study the role of standard PTMs but also of those that are only present in specific histone variants. It makes sense that epigenetic marks involved in the abiotic stress response coincide with stress-specific histone variants in the same nucleosome. An example of specific modifications in histone variants is the ubiquitination of H2A.Z in its K129 residue. This mark specifically controls transcriptional repression by a group of genes silenced by PRC1, suggesting the possible function that the K129Ub might have in the dual role of H2A.Z (Gómez-Zambrano et al., 2019). Moreover, a recent preprint suggest that the K27 residue in the histone variant H3.3 is indispensable for many developmental processes that ranges from flowering to callus formation (Fal et al., 2022). Additionally, it has been reported that the SQ motif present in H2A.W.7 prevents the phosphorylation of the KSPK motif, a mark associated with DNA damage response (Schmücker et al., 2021), which indicates that the differences in the variants’ sequences result in diversity in transcriptional regulation. Consequently, further analyses are needed to fine-tune the relationship between these epigenetic players during the abiotic stress response.

In addition to histone variants and histone modifications, there is a need to unveil the role of DNA methylation, another major epigenetic regulator, in the chromatin landscape of stress-responsive genes. DNA methylation has been broadly described to regulate gene expression and silencing (Robertson, 2005; Slotkin and Martienssen, 2007; Zhang et al., 2018; He et al., 2022). Its relationship in the abiotic stress response as salinity, heat stress, cold, drought, heavy metals or nutrient deficit has been proposed earlier (Villagómez-Aranda et al., 2021; Reddy et al., 2022), although no significant conclusion has been made yet. As this modification colocalizes with heterochromatic regions and transposable elements (TEs) (Henderson and Jacobsen, 2007; Zhang et al., 2018), it makes sense to hypothesize that the CG, CHG and CHH regions of the genome can be methylated and demethylated to alter the transcription of specific stress-responsive genes. So far, it is known that NaCl exposure of *Arabidopsis* DECREASE IN DNA METHYLATION 1 mutant *ddm1*, a chromatin remodeler that facilitates DNA methylation, led to structural chromatin alterations (Yao et al., 2012; Sahu et al., 2013). Also, changes in DNA methylation in response to drought were not only *Arabidopsis* specific but are also observed in rice, which under salt stress showed altered DNA methylation levels (Zhang et al., 2013), maize (Sallam and Moussa, 2021), tomato (Huang et al., 2016), cotton (Wang and Qiao, 2020) and soy (Chen et al., 2019). However, more work needs to be done to explore the in-depth mechanisms and effect of DNA methylation on abiotic stress responses in plants.

To sum up, we consider that the epigenetic changes during the abiotic stress response should not be studied individually but, as the fundamental components of a complex network that provides a regulatory potential. Future insights into how the histone variants and modifications define chromatin organization and impact plant development during the abiotic stress response hold a great potential.

## Author contributions

RN-V contributed most of the writing, that was supervised and revised by BD and CG. All authors contributed to the article and approved the submitted version.
